# Genetic diversity in North American *Cercis Canadensis* reveals an ancient population bottleneck that originated after the last glacial maximum

**DOI:** 10.1038/s41598-021-01020-z

**Published:** 2021-11-08

**Authors:** Meher Ony, William E. Klingeman, John Zobel, Robert N. Trigiano, Matthew Ginzel, Marcin Nowicki, Sarah L. Boggess, Sydney Everhart, Denita Hadziabdic

**Affiliations:** 1 Department of Entomology and Plant Pathology, University of Tennessee, Knoxville, TN USA; 2 Department of Plant Sciences, University of Tennessee, Knoxville, TN USA; 3 Department of Forest Resources, University of Minnesota, St. Paul, MN USA; 4 Department of Entomology, Purdue University, West Lafayette, IN USA; 5 Department of Plant Pathology, University of Nebraska, Lincoln, NE USA

**Keywords:** Biogeography, Community ecology, Evolutionary ecology, Molecular ecology, Population dynamics, Evolutionary genetics, Population genetics, Plant genetics, Population genetics

## Abstract

Understanding of the present-day genetic diversity, population structure, and evolutionary history of tree species can inform resource management and conservation activities, including response to pressures presented by a changing climate. *Cercis canadensis* (Eastern Redbud) is an economically valuable understory tree species native to the United States (U.S.) that is also important for forest ecosystem and wildlife health. Here, we document and explain the population genetics and evolutionary history of this deciduous tree species across its distributed range. In this study, we used twelve microsatellite markers to investigate 691 wild-type trees sampled at 74 collection sites from 23 Eastern U.S. states. High genetic diversity and limited gene flow were revealed in wild, natural stands of *C. canadensis* with populations that are explained by two major genetic clusters. These findings indicate that an ancient population bottleneck occurred coinciding with the last glacial maximum (LGM) in North America. The structure in current populations likely originated from an ancient population in the eastern U.S. that survived LGM and then later diverged into two contemporary clusters. Data suggests that populations have expanded since the last glaciation event from one into several post-glacial refugia that now occupy this species’ current geographic range. Our enhanced understanding benchmarks the genetic variation preserved within this species and can direct future efforts in conservation, and resource utilization of adaptively resilient populations that present the greatest genetic and structural diversity.

## Introduction

The genetic structure and demographics of many North American plant species have been greatly influenced by climate fluctuations that occurred during the Pleistocene epoch^1,2^. During the last glacial maximum (LGM), which occurred approximately 18,000–21,000 years ago^3–5^, the Laurentide Ice Sheet extended from the northernmost portions of North America to 39°N^3^. These events reduced the range of many temperate tree species, forcing them into glacial refugia, which included unglaciated southern regions and suitable micro-environments that were present in northern glaciated regions^1,6^. Many historically contiguous or closely occurring refugia have been identified in the eastern United States (U.S.), but location delineations and number of refugia continue to be debated^6,7^. These refugia are poorly represented within the fossil record, yet the spatial genetic structure and evolutionary histories of many species have been used as evidence of historical refugia^6,8–10^.

According to the “range shift following last glacial maximum” hypothesis, many temperate species recolonized and spread into their current distributions after the LGM^11,12^. The result of this recolonization process can be inferred from the genetic structure within extant plant populations, manifested as reduced genetic diversity along colonization routes, and distinct spatial genetic clusters across the newly expanded range of a species^2,13,14^. Patterns of reduced genetic diversity across a range in expansion are common among European temperate plant species^15^. This trend is not as evident among North American tree species, such as *Carya cordiformis* [Wagenh.] K. Koch (bitternut hickory) and *C. ovata* [Mill.] K.Koch (shagbark hickory), which present relatively uniform genetic variation in their distributions^15^. Genetic homogeneity in North American plant species can be explained by slow post-glacial expansion into new areas, presence of many refugia occurring in close proximity, and high gene flow across time^15,16^. Nevertheless, phylogeographic studies provide evidence that range dynamics of the post-glacial species populations have contributed more to the current patterns of genetic diversity in temperate tree species than any other ecological force (e.g., central- periphery theory^17^, particularly for the populations in the northern edge of a species distribution^11^.

The distribution of tree species, their genetic diversity, and population structure are shaped by many factors including climate oscillations, demographic incidents, ecological and environmental variables, and by their distinct biology^11,18,19^. Yet, the role that glaciation has played in the distribution, range, genetic variation, and spatial genetic structure of outcrossing tree species that span a geographically wide range are not well understood, especially across the eastern U.S. To better understand the role of LGM in structuring current species distributions and population structure of temperate tree species in the eastern U.S., we evaluated the spatial population structure of widely distributed forest understory tree *Cercis canadensis* L. (*C. canadensis* var. *canadensis* L.; Fabaceae; eastern redbud). *Cercis canadensis* is a great system to study the role of LGM in eastern U.S. tree species, due to its wide and continuous geographic distribution without any major geographical barriers across eastern U.S.

*Cercis canadensis* is a self-incompatible^20^, deciduous tree native to the midwestern and eastern U.S., as well as northeastern Mexico^21,22^. This species grows well in partial shade, is well adapted to a wide-range of climate conditions and elevations, and can be found in the USDA hardiness zones 4 through 9^23,24^. This relatively small ornamental tree is characterized by its wide, colorful, umbrella-shaped canopy^25^, and is a popular landscape tree due to its heart-shaped foliage, compact form, and early spring flowers^23^.

When a fine-scale, smaller, and fragmented population of *C. canadensis* was examined with microsatellite loci^26^, wild trees maintained high genetic diversity, gene flow, and moderate to high genetic differentiation^27^. Although *C. canadensis* is ecologically important, there is limited knowledge of the contemporary genetic diversity, spatio-temporal genetic structure, gene flow, and past evolutionary history of this species across its native range in the U.S. To address this knowledge gap, we used microsatellite loci to accomplish the following: (1) characterize the genetic diversity of the wild populations of *C. canadensis* within its native range in the U.S.; (2) infer patterns in the spatial genetic structure of *C. canadensis*; and (3) reveal the evolutionary demographics in its native range. We hypothesized that *C. canadensis* wild populations will be genetically diverse and genotypes will be spatially clustered across its native range. We also hypothesized that the genetic structure would be consistent with range expansion that occurred from one of several southern glacial refugia. More specifically, we aimed to explore the following questions: (1) Do genetic diversity and population structures patterns reflect evidence for northern glacial refugium? We expected to detect high genetic diversity with distinct population structure in northern range limit, otherwise a trend of high to low genetic diversity from south to north would indicate recolonization of *C. canadensis* in north from one or more southern glacial refugia; (2) Is there any evidence for micro-refugia in current *C. canadensis* species distribution range? If there were multiple micro-refugia present in close proximity, especially in southern region, we expected to detect genetic homogeneity in the studied populations.

## Materials and methods

### Sample collection

Leaf samples of *C. canadensis* were collected by authors, collaborators, and citizen scientists (*see acknowledgements*), who sampled specimens across the native range of this species in 23 states in the midwestern and eastern U.S. (Table 1). The use of trees in the present study complied with international, national and/or institutional guidelines. Plants were identified based on collection guide provided to our collectors and confirmed by co-authors (voucher specimen deposited at the University of Tennessee Vascular Herbarium, catalog # TENN-V-0246136). For each collection site, at least 10 non-cultivated *C. canadensis* trees occurring within a one-mile radius were selected and their geographical coordinates were recorded. From each tree, five to seven young and disease-free leaves were collected, held between several pieces of absorbent paper and stored at ambient room temperature in a paper envelope until processing. Leaf samples from 1193 individual trees were collected at 117 collection sites. To avoid the over-representation of trees within a geographical area, we randomly selected a subset of collection sites from geographical areas with more than one collection site sampled. This study used total of 790 trees representing 79 collection sites that span much of the current native geographic range of *C. canadensis.*

**Table 1:** Genetic diversity indices across 74 *Cercis canadensis* collection sites from the United States using 12 microsatellite loci.

State	County name	Site acronym	Latitude	Longitude	N	MLG	H_E_	rbard	*P* value	Pa
Alabama	Jefferson	AL1	33.7197	− 86.7748	10	10	0.51	0.27	0.01	1
Alabama	Jefferson	AL2	33.5670	− 86.6802	10	10	0.45	0.31	0.01	0
Alabama	Tallapoosa	AL3	32.7705	− 85.7854	10	9	0.42	0.35	0.01	0
Arkansas	Pulaski	AR1	34.7601	− 92.3173	10	10	0.48	0.02	0.22	0
Arkansas	Washington	AR2	36.0878	− 94.1679	10	10	0.52	0.03	0.14	0
Arkansas	Washington	AR3	36.1316	− 94.1336	10	10	0.50	0.03	0.13	0
Florida	Gadsden	FL1	30.6264	− 84.8949	9	9	0.33	0.10	0.02	0
Florida	Alachua	FL2	29.7761	− 82.5081	10	10	0.56	0.24	0.01	1
Florida	Dixie	FL3	29.6367	− 82.7913	10	10	0.52	0.24	0.01	0
Georgia	Whitfield	GA1	34.7287	− 85.0314	10	10	0.53	0.26	0.01	0
Georgia	Clarke	GA2	33.8804	− 83.3572	10	10	0.35	0.10	0.02	0
Georgia	Talbot	GA3	32.6655	− 84.5126	10	10	0.34	0.04	0.11	0
Georgia	Clarke	GA4	33.9032	− 83.3869	10	10	0.48	0.06	0.36	0
Iowa	Fremont	IA2	40.6746	− 95.6908	8	8	0.64	0.10	0.02	1
Indiana	Jefferson	IN1	38.7831	− 85.3695	6	6	0.64	0.09	0.37	1
Indiana	Tippecanoe	IN2	40.5306	− 86.9244	10	10	0.60	0.04	0.36	1
Indiana	Montgomery	IN3	40.0494	− 86.9016	9	9	0.57	0.00	0.76	0
Indiana	Parke	IN4	39.8820	− 87.2020	10	10	0.62	0.03	0.52	0
Kansas	Geary	KS1	38.8941	− 96.8544	10	10	0.56	0.05	0.04	0
Kansas	Butler	KS2	38.8681	− 96.8501	10	10	0.52	0.03	0.12	0
Kentucky	Laurel	KY1	37.2145	− 84.1943	7	7	0.51	0.21	0.01	0
Kentucky	Madison	KY2	37.8949	− 84.2743	10	10	0.46	0.01	0.29	2
Kentucky	Bath	KY3	38.1049	− 83.8265	10	10	0.59	0.10	0.01	0
Kentucky	Carter	KY4	38.3641	− 82.8029	8	8	0.48	0.19	0.01	0
Maryland	Carroll	MD1	39.3548	− 76.8963	10	10	0.51	0.07	0.02	0
Maryland	Prince George	MD2	38.7473	− 76.9924	6	6	0.56	0.45	0.01	0
Michigan	Washtenaw	MI1	42.3828	− 83.9068	7	7	0.47	0.06	0.05	0
Michigan	Berrien	MI2	41.9052	− 86.3701	9	9	0.63	0.01	0.93	0
Michigan	Berrien	MI3	41.7903	− 86.7627	9	9	0.66	0.07	0.01	0
Missouri	Boone	MO1	38.8401	− 92.2923	10	10	0.59	0.03	0.12	0
Missouri	Genevieve	MO2	38.0137	− 90.2203	10	10	0.54	0.07	0.01	0
Missouri	Boone	MO3	39.0788	− 92.3066	10	10	0.52	0.11	0.01	1
Missouri	Dent	MO4	37.4552	− 91.6869	10	10	0.52	0.06	0.06	0
Mississippi	Pontotoc	MS2	34.1447	− 88.9972	10	10	0.53	0.13	0.01	0
Mississippi	Oktibbeha	MS3	33.4258	− 88.7004	10	10	0.53	0.24	0.01	1
North Carolina	Guilford	NC1	36.0322	− 79.7063	8	8	0.51	0.03	0.43	0
North Carolina	Cabarrus	NC2	35.4578	− 80.5947	9	9	0.47	− 0.01	0.79	1
North Carolina	Mecklenburg	NC3	35.2415	− 80.9845	10	10	0.47	0.18	0.01	0
North Carolina	Wake	NC4	35.9789	− 78.6368	10	10	0.58	0.03	0.06	0
Nebraska	Sarpy	NE1	41.1799	− 95.9181	10	10	0.68	0.02	0.32	0
New York	Monroe	NY1	43.0339	− 77.5503	10	10	0.53	0.35	0.01	0
New York	Tompkins	NY2	42.3988	− 76.5549	9	9	0.61	0.24	0.01	0
Ohio	Hamilton	OH1	39.0857	− 84.3589	9	9	0.67	0.13	0.01	0
Ohio	Hamilton	OH2	39.0390	− 84.3478	10	10	0.63	0.07	0.01	1
Oklahoma	Cherokee	OK2	35.7603	− 94.9070	10	10	0.54	0.05	0.02	0
Pennsylvania	Centre	PA1	40.8038	− 77.8058	10	10	0.58	0.25	0.01	0
Pennsylvania	Huntingdon	PA2	40.5064	− 77.9806	9	9	0.47	0.20	0.01	0
Pennsylvania	Juniata	PA3	40.5658	− 77.2557	6	6	0.52	− 0.02	0.49	0
South carolina	Pickens	SC1	34.6415	− 82.8241	10	10	0.57	0.11	0.01	0
South carolina	Lexington	SC2	34.1716	− 81.3047	8	8	0.56	0.03	0.75	0
Tennessee	Cocke	TN1	35.8211	− 83.1528	8	8	0.46	0.12	0.02	0
Tennessee	Anderson	TN10	36.0054	− 84.2077	10	10	0.32	0.05	0.13	0
Tennessee	Knox	TN11	35.9462	− 83.9178	9	9	0.38	0.00	0.54	0
Tennessee	Hamilton	TN2	35.0758	− 85.1288	10	10	0.50	0.02	0.26	0
Tennessee	Hamilton	TN3	35.0235	− 85.3791	10	10	0.54	0.04	0.11	0
Tennessee	Shelby	TN4	35.0892	− 89.8661	10	10	0.47	0.31	0.01	0
Tennessee	Shelby	TN5	35.2723	− 89.6452	10	10	0.36	0.19	0.01	0
Tennessee	Shelby	TN6	35.3255	− 90.0515	10	10	0.55	0.03	0.12	0
Tennessee	Cheatham	TN7	36.1222	− 87.1416	10	10	0.52	− 0.02	0.70	0
Tennessee	Wilson	TN8	36.0564	− 86.4258	10	10	0.55	0.15	0.01	1
Tennessee	Wilson	TN9	36.1683	− 86.5654	10	9	0.44	0.13	0.01	0
Texas	Sabine	TX1	31.4106	− 94.0225	10	10	0.44	0.02	0.25	1
Texas	Smith	TX2	32.4520	− 95.2534	10	10	0.55	− 0.03	0.92	2
Texas	Collin	TX3	33.0794	− 96.5488	9	9	0.51	0.02	0.25	0
Virginia	Prince William	VA1	38.8121	− 77.5524	9	9	0.52	0.10	0.04	0
Virginia	King George	VA2	38.3293	− 77.0908	7	7	0.57	0.06	0.42	1
Virginia	Brunswick	VA3	36.9225	− 77.7582	10	10	0.55	0.06	0.03	0
Virginia	Washington	VA4	36.6563	− 81.9050	10	10	0.55	0.32	0.01	0
Virginia	Radford	VA5	37.1345	− 80.5218	10	10	0.47	0.38	0.01	0
West Virginia	Cabell	WV1	38.3925	− 82.4245	8	8	0.59	0.12	0.01	0
West Virginia	Kanawha	WV2	38.4842	− 81.4354	10	10	0.56	0.20	0.01	1
West Virginia	Braxton	WV3	38.8735	− 80.6293	8	8	0.48	− 0.01	0.70	1
West Virginia	Monongalia	WV4	39.6034	− 79.9922	7	7	0.50	0.11	0.03	0
**Average/total**					**691**	**689**	**0.67**	**0.05**	**0.01**	**19**

N = total number of samples per collection site, MLG = number of diploid individuals multilocus genotypes after clone correction, H_E_ = Nei's genotypic diversity corrected for sample size, rbard = linkage disequilibrium, Pa = number of private alleles in each collection site.

### DNA extraction

From each tree, 60 to 100 mg of dry leaf tissue was used to isolate DNA. Samples were homogenized four times for 30 s each at 6 m/s using a Beadmill 24 homogenizer (Fisher Scientific, Pittsburgh, Pennsylvania, U.S.) and were kept in liquid nitrogen for 5 min between each pulverization step. The Qiagen DNeasy Plant Mini Kit (Qiagen, Valencia, California, U.S.) was used to isolate genomic DNA (gDNA) from the pulverized samples with the following minor modifications in the manufacturer’s provided protocol. Specifically, 2% w/v polyvinylpyrrolidone (PVP) was mixed into the lysis buffer (AP1). Then 8 µl of RNase was added into each sample tube and incubated at 65 °C in a water bath for 45 min. Every two mins each sample tube was inverted gently to mix the sample well. Lastly, samples were incubated at − 20 °C for at least one hour. Ethanol was used to wash the spin columns if there was any visible remaining debris and elution buffer added. Elution buffer was heated to 65 °C before 50 µl was added to the spin columns twice. Concentrations of gDNA were quantified using ND1000 Ultraviolet-Vis Spectrophotometer (NanoDrop Technologies, Wilmington, Delaware, U.S.) and the gDNA was stored at − 20 °C until further use.

### Microsatellite primers and genotyping conditions

Initially, gDNA was isolated from five *C. canadensis* individuals from the University of Tennessee Gardens (Knoxville, Tennessee, U.S.) and used to evaluate 68 candidate microsatellite loci^26^. Primers for twelve polymorphic microsatellite loci (Table 2) were selected for this study based on the successful amplification and PCR product size agreement with the published data. Microsatellite loci were amplified with polymerase chain reaction (PCR) in a 10 µl reaction mixture containing the following: 1 µl gDNA, 1 µl of 10 µM of each forward and reverse primer, 0.5 µl of dimethyl sulfoxide, 4 µl of GoTaq G2 Hot Start Master Mix (Promega Corp, Madison, Wisconsin U.S.), and 2.5 µl sterile molecular grade water. To assure validity of the data, both a negative control (reaction mixture with water instead of any DNA sample) and a positive control (a DNA sample from the initial screens that amplified across all microsatellite primers) were incorporated for every primer-pair tested. Amplification of DNA with 12 microsatellite loci across all samples was completed in 96 well plates using an Eppendorf Thermocycler (Eppendorf AG, Hamburg, Germany) with the following thermal profile: initial denaturation at 94 °C for 3 min, followed by 35 cycles of denaturation at 94 °C for 30 s, annealing at 55 °C for 30 s, and an extension at 72 °C for 30 s, with a final extension of 72 °C for 4 min.

**Table 2:** Genetic diversity indices of 12 microsatellite loci across 74 collection sites of *Cercis canadensis*.

Locus	Repeat motif	Allele No	Ar	H_O_	H_E_	H	Evenness	*F*_ST_	*F'*_ST_	*F*_IS_	Nm	Pa
127spa	(TC)4	12	1.73	0.99	0.75	1.44	0.64	0.05	0.05	− 0.39	0.82	1
168a	(CT)7	13	1.51	0.23	0.70	1.45	0.69	0.24	0.24	0.57	0.58	4
177b	(GA)6	9	1.64	0.23	0.78	1.70	0.80	0.15	0.15	0.66	0.84	0
199a	(GAGA)8	12	1.55	0.23	0.71	1.58	0.65	0.21	0.21	0.59	0.65	1
220a	(TATT)4	11	1.64	0.57	0.80	1.66	0.64	0.19	0.20	0.11	0.70	2
229a	(GAGAG)4	6	1.50	0.15	0.62	1.11	0.77	0.15	0.15	0.71	0.86	0
625a	(GA)4	11	1.57	0.24	0.68	1.32	0.59	0.13	0.13	0.59	0.81	2
658a	(CT)6	9	1.50	0.21	0.60	1.17	0.69	0.13	0.13	0.59	0.89	3
680a	(GT)8	8	1.37	0.01	0.59	1.15	0.66	0.33	0.33	0.98	0.37	0
762a	(TC)7	11	1.52	0.28	0.68	1.41	0.69	0.21	0.21	0.48	0.64	3
780b	(AG)12	16	1.79	0.70	0.86	2.19	0.75	0.07	0.07	0.12	1.70	1
995a	(AG)7	7	1.23	0.01	0.52	0.84	0.82	0.53	0.54	0.99	0.18	2
**Average/Total**		**10**	**1.55**	**0.32**	**0.69**	**1.42**	**0.70**	**0.19**	**0.19**	**0.43**	**0.75**	**19**

Ar = allelic richness corrected for sample size, H_O_ = observed heterozygosity, H_E_ = Nei's genotypic diversity, H = Shannon–Wiener index, F_ST_ = populations fixation index, F'_ST_ = population differentiation, F_IS_ = inbreeding coefficient, Nm = gene flow, Pa = number of private alleles in each collection site.

Amplified PCR products were visualized with QIAxcel Capillary Electrophoresis System (Qiagen) and analyzed with a 15/600 bp internal alignment marker and a 25 to 500 bp DNA ladder. All *C. canadensis* gDNA samples were amplified and visualized against each of the 12 microsatellite loci using the procedure described above. Reactions not producing any amplified products were rerun once before they were considered missing data. Samples with ≥ 40% missing data were discarded. Also, collection sites with more than four samples having ≥ 40% missing data were excluded from the dataset.

### Genetic diversity

Using the Excel macro FLEXBIN version^28^, raw allele sizes were converted into allelic classes. In this program, alleles were binned into base-pair (bp) size categories by statistical similarities. This binned genetic dataset was used for all of the following statistical analyses, which were completed using R version 3.5.3^29^. Clone-correction of the data was implemented to identify presence of clonal individuals at the collection site level using the R package POPPR version 2.8.2^30,31^. For each collection site, only multi-locus genotypes (MLG) were used to obtain unbiased estimates of allelic frequency from the dataset^32^.

R package POPPR was used to calculate the total number of alleles per locus, observed heterozygosity (H_O_; number of the heterozygotes present at a locus which is divided by sample size), expected heterozygosity (H_E_; calculated as expected heterozygosity per locus^33^), and linkage disequilibrium (rbard); non-random association of alleles between loci). Additionally, the Shannon-Weiner diversity index (H) was calculated for each collection site using POPPR. H considers both allele richness and evenness of the allelic distribution^34^. The number of unique private alleles in collection sites and different loci was estimated in POPPR package. Allelic richness (Ar), a measure of rarefied allelic counts per locus, was estimated using package HIERFSTAT version 0.04–22^35^. Allelic richness is used as an estimate of the long-term evolutionary potential to adapt and persist in a given population^36,37^. The genetic fixation index (*F*_ST_), inbreeding coefficient (*F*_IS_), and allelic differentiation (*F*’_ST_)^38,39^ were calculated using HIERFSTAT package. Gene flow (Nm) was estimated using GenAlEx 6.5 software (Peakall & Smouse, 2006; Peakall & Smouse, 2012). In the program, Nm was estimated as the effective number of the migrants per locus based on F-statistics.

### Population structure

Population structure within the native range of wild *C. canadensis* trees was analyzed using the program STRUCTURE version 2.3.4^40^ to which an admixture model was applied. This Bayesian clustering method with Monte Carlo Markov Chain (MCMC) approach was used with the following parameters: 500,000 burn-in period with 1,000,000 MCMC repetitions for 30 independent chains for *K* values from 1 to 18. The resulting output was visualized with STRUCTURE HARVESTER web version 6.94^41^. The optimum *K* value, indicator of population clusters present in the dataset, was calculated utilizing the Evanno method^42^. The estimation of *ΔK* criterion obtained from STRUCTURE HARVESTER were visualized using POPHELPER 2.2.6^43^ that merged the 30 independent chains. R packages MAPS version 3.3.0^44^ and PLOTRIX version 3.8–1^45^ were used to generate pie charts of admixture proportions at K = 2.

Several model-free methods were utilized to investigate the population structure of *C. canadensis* samples. A Neighbor-Joining (NJ) dendrogram was constructed using Nei’s genetic distance in POPPR^46,47^. Discriminant Analysis of Principal Components (DAPC) was implemented using package ADEGENET version 2.1.1^48^ to visualize the underlying genetic structure of this species in its wide geographical range. This is a two-step multivariate analysis that investigates the genetic variations within populations among the sampled collection sites^49^. At first, a principal component analysis (PCA) was conducted, and then the number of PCA vectors (to explain majority of variance with minimizing over-fit of the DAPC) was selected. Then, a selected number of PCAs were used to reveal differences between groups while minimizing within group variations, as well as ordination of collection sites into distinct groups using discriminant analysis^49,50^. Moreover, missing values were calculated as mean allele frequency and cross-validation analysis was performed to select appropriate PC numbers.

Isolation by distance (IBD) was estimated using the Mantel test^51,52^ with 10,000 permutations in package VEGAN version 2.5–6^53^ using Euclidean distance. IBD checks for a correlation between genetic distance and geographical distance among the individuals in a dataset. The Mantel test was implemented across the 74 collection sites while considering the whole dataset as one population.

Analysis of Molecular Variance (AMOVA)^54^ was carried out using POPPR with 10,000 permutations by sorting the individuals into hierarchical groups to assess the degree of molecular variance partitioned within, between, and among the collection sites. The levels of population hierarchy included: (1) 74 collection sites as one hierarchical group; (2) two groups on the basis of the STRUCTURE analysis; and (3) four major groups on the basis of five major eco-region divisions namely hot continental division (mountain provinces), hot continental division, warm continental division, subtropical division, and prairie division (see Supporting Information Fig. S2) in the midwestern and eastern U.S. (Bailey, 1994). *C. canadensis* collection sites in warm continental division were grouped with hot continental division collection sites. As *C. canadensis* is found in wide range of climate and elevations, we tested if there was any influence of the regional climate patterns in the population structures of *C. canadensis* in AMOVA analysis.

### Demographic histories

To investigate and interpret the evolutionary history of *C. canadensis*, we used DIYABC program version 2.1^55,56^ that utilized Approximate Bayesian Computation (ABC) statistical methods. For this analysis, collected individuals were pooled into two major groups, based on the STRUCTURE results. To elucidate the evolutionary history of *C. canadensis*, we analyzed competing scenarios in two ABC steps. In the first step, we tested five demographic scenarios using 200,000 simulated pseudo-observed datasets (PODs) wherein: (1) the first two scenarios suggested stepwise divergence of the current two major groups from an ancient population, (2) a third scenario suggested a single, two-way split of contemporary groups from an ancient unsampled population, and (3) the last two scenarios were based on the hypothesis of divergence of current groups from two separate ancient un-sampled populations. Once the analysis of these scenarios was completed, the two scenarios from the first step that yielded higher logistic regression support were selected as the basis for assessing the second step of ABC. In the second step, seven scenarios were constructed that addressed the possibility of a bottleneck occurrence within the evolutionary history of the species. Over 1,000,000 pseudo-observed datasets (PODs) were simulated under the assumed prior parameter ranges for each scenario. Posterior probabilities of the compared scenarios were estimated to select the best supported scenario^55^.

## Results

### Microsatellite genetic diversity and hierarchical fixation indices

Twelve microsatellite loci were amplified from 790 *C. canadensis* trees sampled in this study. Due to presence of missing data (missing ≥ 40% SSRs), five of 79 collection sites were excluded and 49 individuals from the remaining 74 sites were discarded, resulting in 691 individuals from 74 collections. Additionally, after deleting two clonal individuals, 689 unique multilocus genotypes from 74 collection sites remained for further data analyses (Table 1). The average null alleles or missing data across the dataset were overall 2.89% (see Supporting Information Fig. S1). Nei’s genetic diversity index (H_E_) value in the studied 74 collection sites was 0.67, ranging from 0.32 (Anderson county, TN10) to 0.68 (Sarpy county, NE1) (Table 1). Moreover, weak (rbard = 0.05, *P* value = 0.01) but significant linkage disequilibrium value detected in the dataset. Nineteen private alleles were detected within the 74 collection sites (Table 1). In addition, 9 of the 12 microsatellite loci yielded private alleles, and the highest number of private alleles was recovered from locus 168a (Pa = 4, Table 2). The number of alleles per locus ranged from 6 to 13 with a mean of 10 alleles per locus (Table 2). Overall allelic richness (Ar) ranged from 1.23 for locus 995a to 1.79 for locus 780b, with a mean of 1.55, implying a presence of high allelic richness in wild *C. canadensis* individuals. Observed heterozygosity (H_O_) across all loci was 0.32, ranging from 0.01 (locus 995a and 680a) to 0.99 (locus 127spa). The overall expected heterozygosity (H_E_) across all 12 microsatellite loci was high (H_E_ = 0.69), ranging from 0.52 (locus 995a) to 0.86 (locus 780b).

The overall Shannon-Weiner diversity index (H) for the 12 loci was 1.42 and ranged from 0.84 (locus 995a) to 2.19 (locus 780b; Table 2). Additionally, high population fixation (*F*_ST_ = 0.19; ranging from 0.05 to 0.53; Table 2) and population differentiation (*F*’_ST_ = 0.19; ranging from 0.05 to 0.54) were identified among *C. canadensis* populations. We estimated an inbreeding coefficient (*F*_IS_) of 0.43 across all loci, indicating excess homozygotes (Table 2) among the studied *C. canadensis* populations. The average estimated gene flow was 0.75, which indicates that a limited amount of gene flow has occurred among the studied populations (Table 2).

### Population structure

**Figure 1:**
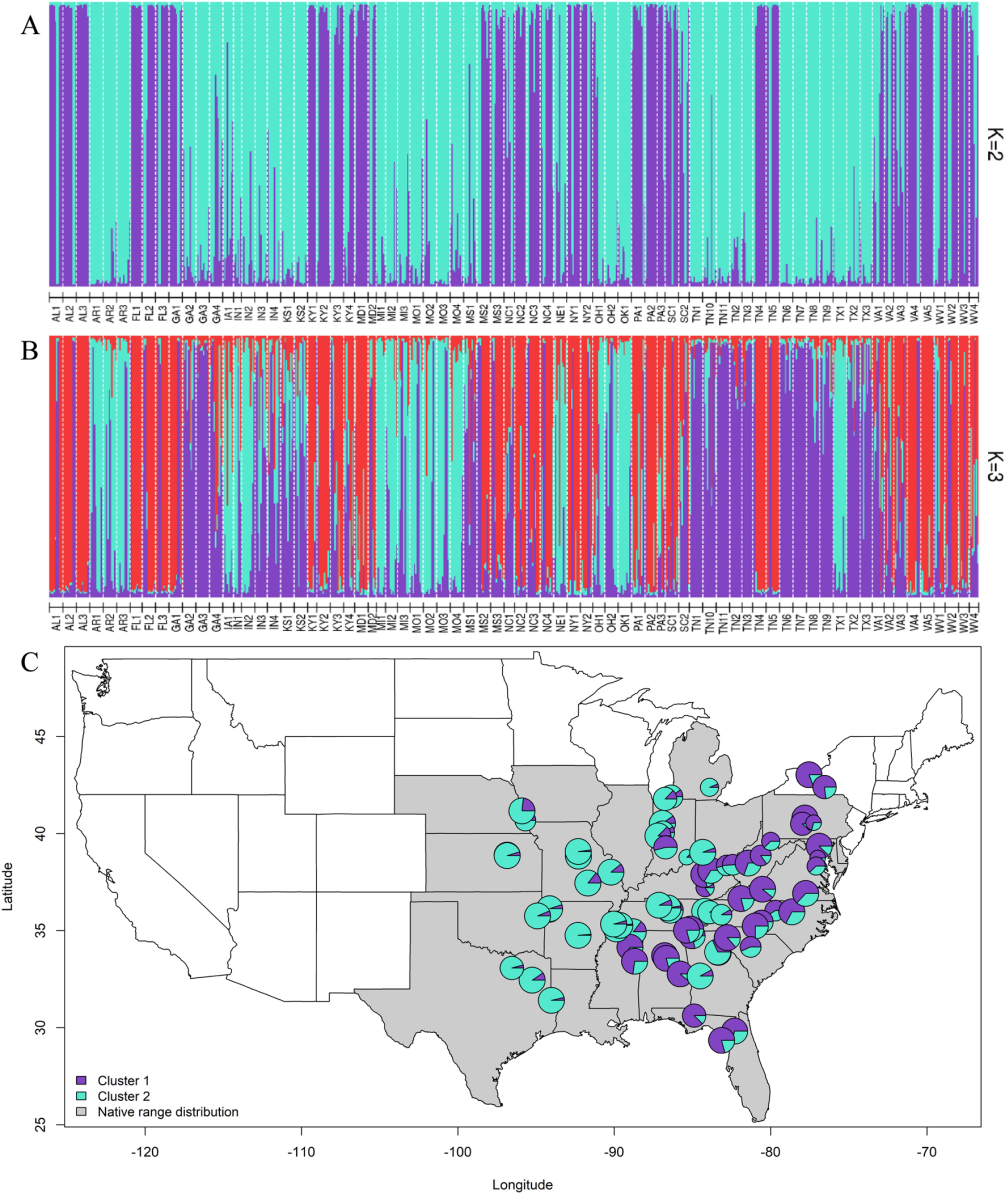
**(A**, **B**, **C)**. STRUCTURE bar graphs representing genetic clusters (*ΔK* = 2–3) among 74 collection sites of *Cercis canadensis* (**A** and **B**). Each vertical bar represents an individual sample and the color of the bar indicates the assignment probability of that individual to belong to one of the identified clusters. Pie charts of admixture coefficients inferred by STRUCTURE (*ΔK* = 2), plotted across geographical distribution of *C. canadensis* in the eastern United States (C) and 74 collection sites used in this study.

**Figure 2:**
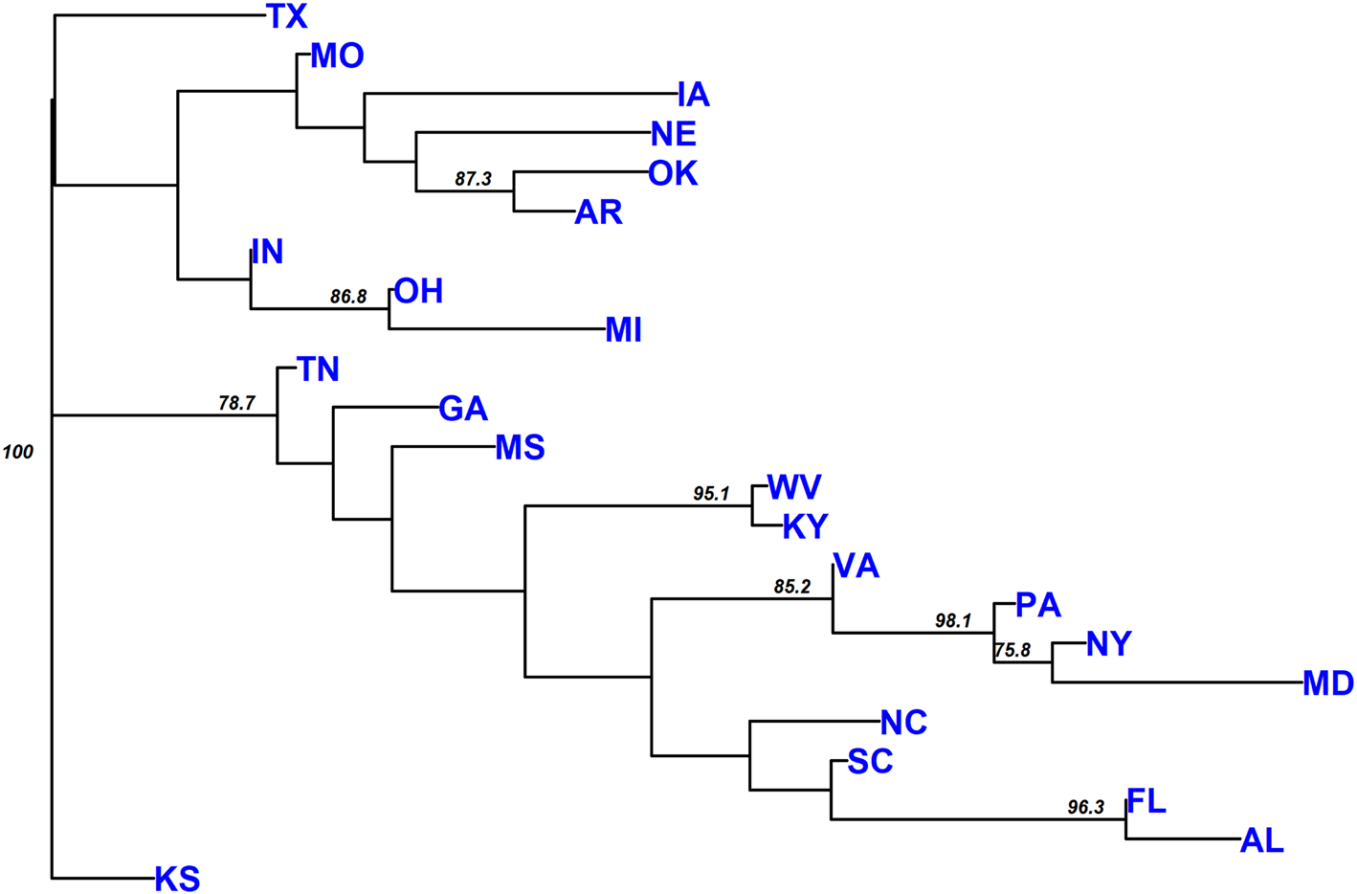
Neighbor-joining tree of 74 collection sites of *Cercis canadensis* using Nei's genetic distance. Numbers indicate the percentage of bootstrap support using 1,000 replications (threshold set at 70%).

**Figure 3:**
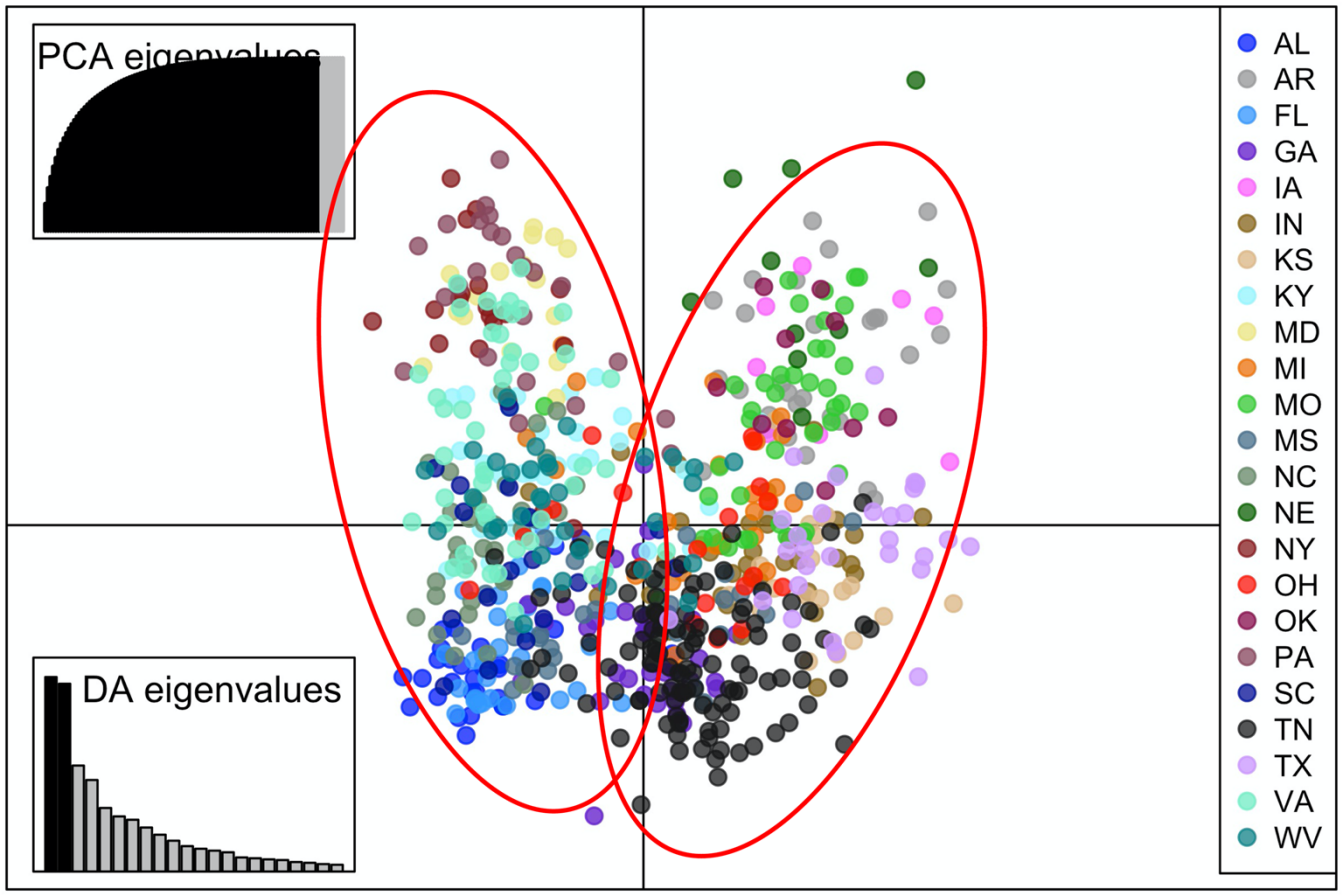
Discriminant Analysis of Principal Components (DAPC) plot of *Cercis canadensis* individuals across 23 states in e U.S. The first 47 principal components explained 94% of the variation in *C. canadensis* individuals in the dataset. Here, allele 154 at locus 199a explained 12% of the variance and allele 102 at locus 220a explained 13% of the variance on the first axis (threshold = 0.06). Data was constructed using 1,000 permutations. Discriminant Analysis eigenvalues are also presented.

Using Nei’s genetic distance, we estimated pairwise *F*_ST_ values among the 74 collection sites and the values ranged from 0.02 to 0.33 (see Supporting Information Table S1). STRUCTURE results revealed an optimum *∆K* = 2, implying that across its wide native range, *C. canadensis* collection sites are divided into two major clusters. Collection sites in the northern-most collection region of the U.S. (Ohio to Nebraska) and mid-south to mid-north (from Texas to Nebraska) were part of the first cluster (designated as north genetic cluster) (Fig. 1). The rest of the collection sites from the northeast (New York) to mid-south (Mississippi) along the Atlantic Ocean coastline belonged to the second cluster (designated as south genetic cluster). Whereas, a constructed NJ dendrogram revealed the presence of two major groups (except KS and TX collection sites that did not group with any major group), which supported the STRUCTURE findings of presence of two genetic clusters (Fig. 2). In addition, the collection site distribution in these two major groups (NJ dendrogram) is similar to the distribution of collection sites in the two STRUCTURE-based clusters. The DAPC biplot further confirmed the presence of genetic structures, primarily along the x-axis with two overlapping clusters (Fig. 3). Therefore, based on additional analyses used in this study, the grouping of *C. canadensis* individuals is best explained with two genetic clusters (Fig. 1–3). These analyses also showed that the majority of the collection sites (except two collection sites from Georgia) grouped in clusters based on their geographical origin.

In the Analysis of Molecular Variance (AMOVA) analysis, the first data arrangement showed that most of the genetic variation was present within 74 collection sites (74.2%, *P* < 0.001) (Table 3). A significant amount of variation was also partitioned among collection sites (25.8%, *P* < 0.001). When the dataset was divided into two genetic clusters based on STRUCTURE results, 69.2% (*P* < 0.001) of the genetic variability was attributed to the location of collection sites (Table 3). There was also a significant amount of variability between the two different genetic clusters (13.9%, *P* < 0.001) and between collection sites within the clusters (17%, *P* < 0.001) (Table 3). When the dataset was partitioned by the major eco-regions groups that are represented across the distribution of *C. canadensis*, only 7.9% (*P* < 0.001) of the variability could be attributed among eco-region groups, versus 18.6% (*P* < 0.001) variability that was attributed among the collection sites within groups (Table 3). The majority of genetic variation was explained among individuals within a collection site, rather than among populations or group levels for all three tested scenarios (Table 3). Nevertheless, the extent of variation that was observed within collection sites and between clusters revealed the presence of genetic structure. AMOVA results were, therefore, congruent with the hierarchical fixation indices and indicated the presence of population structure. However, the lowest amount of variation was found within the groups when the data were divided according to major eco-regions. This finding suggests that partitioning trees within eco-regions cannot be expected to explain the genetic differentiation and population structure observed in *C. canadensis* wild populations. Results from the isolation-by-distance analysis indicated that among *C. canadensis* populations, the geographical distance effect was weak, but linearly correlated (r = 0.08, *P* < 0.001) with genetic distance (see Supporting Information Fig. S2).

**Table 3:** Analysis of Molecular Variance of *Cercis canadensis* across 12 microsatellite loci for (i) 74 collection sites as one hierarchical cluster, (ii) 74 collection sites divided into two groups (north and south) based on STRUCTURE results (two clusters), and (iii) four eco-regions.

Source of variation	df	Sum Square	Variance Component	% of Variation	*P* value
(i) 74 collection sites					
Between 74 collection sites	73	1441.17	1.72	25.76	0.001
Within 74 collection sites	561	2785.17	4.96	74.24	0.001
Total	634	4226.35	6.69	100.00	
*F*_ST_ = 0.26					
(ii) Two clusters (STRUCTURE)					
Between two clusters	1	331.78	1.00	13.87	0.001
Between collection sites	72	1109.40	1.22	16.97	0.001
Within collection sites	561	2785.17	4.96	69.15	0.001
Total	634	4226.34	7.18	100.00	
*F*_ST_ = 0.31, *F*_IS_ = 0.20, *F*_CT_ = 0.14					
(iii) Four eco-regions					
Between four eco-regions	3	320.78	0.55	7.88	0.001
Between collection sites within eco-regions	71	1192.76	1.30	18.55	0.001
Within collection sites	600	3087.95	5.15	73.57	0.001
Total	674	4601.49	7.00	100.00	
*F*_ST_ = 0.26, *F*_IS_ = 0.20, *F*_CT_ = 0.08					

F_ST_ = variance among collection sites relative to the total variance.

F_IS_ = inbreeding coefficient of individuals relative to population.

F_CT_ = variance among groups relative to the total variance.

### Demographic histories

**Figure 4:**
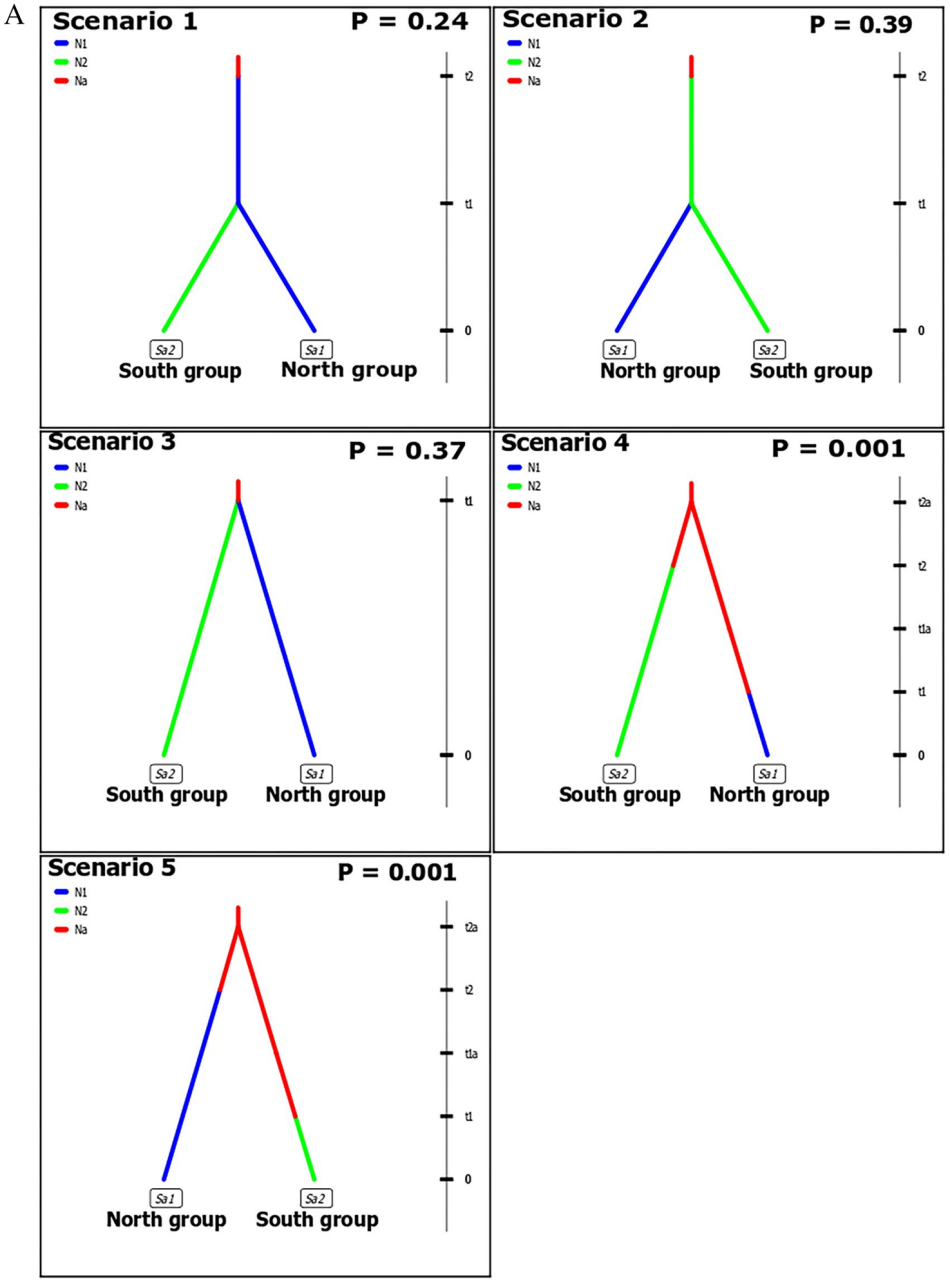

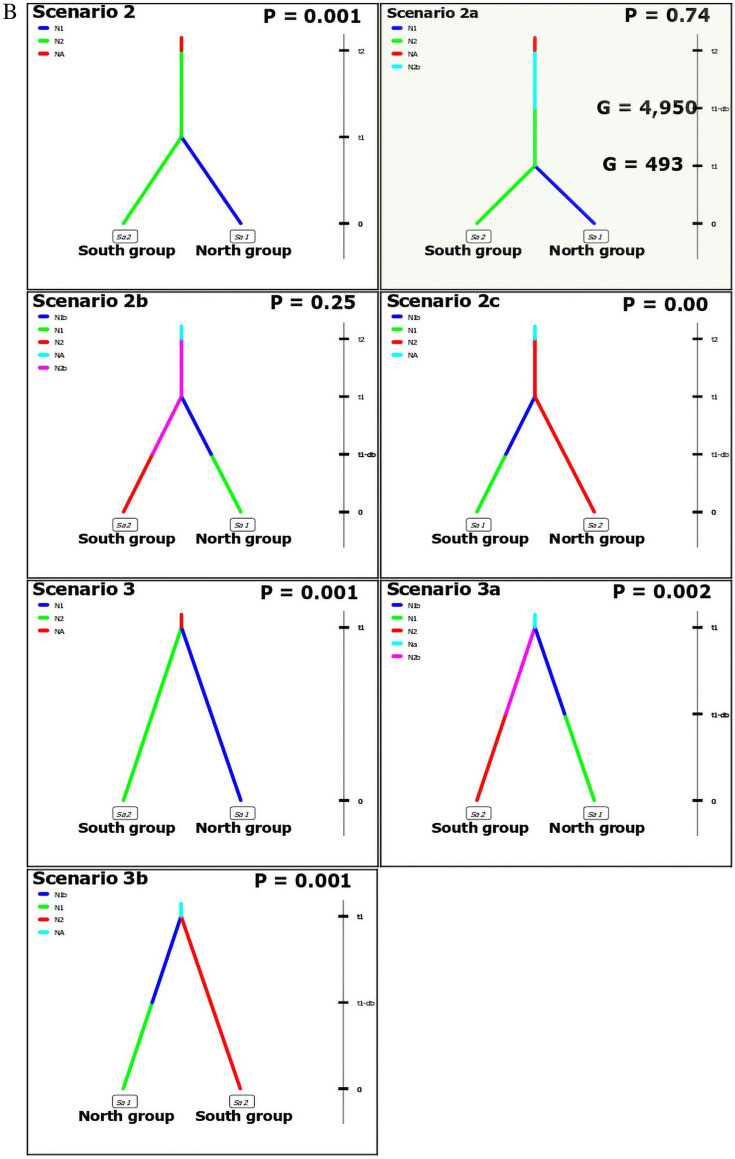
(**A**, **B**)**.** Probable DIYABC evolutionary scenarios for *Cercis canadensis* evolutionary history. Here, current *C. canadensis* populations are north (N1) and south (N2). Also, N1b and N2b represent the populations of N1 and N2 before bottleneck event. On the right side of each scenario, a time scale indicated the timeline of each event (t = 0 is current time, t1-db = bottleneck occurrence, t1/t2 = split of the populations from originating population). Our data was analyzed using two ABC steps resulting in five competing scenarios from the first step (4A), and seven scenarios with possible bottleneck events from the second step analyses (4B). The scenario 2a (B) from step 2 gained the most support in DIYABC analysis and the timeline for the bottleneck (t1-db) and divergence (t1) events of scenario 2a is given in generations. For each scenario, value of relative posterior probability (P) was reported.

The DIYABC program with the ABC approach, however, supported the presence of population structure and found evidence for an ancient bottleneck event occurring in *C. canadensis* wild populations. From the first step of the analysis, two probable scenarios were chosen according to their posterior relative support (Scenario 2, posterior probability (P) = 0.39 and Scenario 3, posterior probability (P) = 0.37; Fig. 4A). In these analyses, Scenario 2 provided evidence that the contemporary *C. canadensis* population originated in the southeastern U.S. region from an ancient population and then later, a north population group (first genetic cluster) diverged from the south population group (second genetic cluster). Alternatively, Scenario 3 suggested that both current *C. canadensis* groups (north and south) have split from an ancient, as yet unsampled group (Fig. 4A). In the second step of the ABC analysis (Fig. 4B), the principal component analysis and relative posterior probability tests revealed that Scenario 2a (posterior probability (P) = 0.74, Fig. 4B) was the most supported and therefore had the greatest likelihood of accurately describing the evolutionary processes that are evident within native stands of *C. canadensis*. Thus, we infer from Scenario 2a that from an ancient population, a group of *C. canadensis* in the south first endured a bottleneck, and then later, a group of northern *C. canadensis* diverged from the southern group (Scenario 2a, Fig. 4B).

Estimated posterior parameters of Scenario 2a suggested that a population bottleneck occurred approximately 4,950 generations ago (ranging from 722 to 9,650 generations in simulated datasets), which is approximately 25,000 years ago, given the average time for a *C. canadensis* tree to reach reproductive maturity is six to seven years^57^ (Fig. 4B). Therefore, the bottleneck event most probably occurred during the last glacial period, which ended about 21,000 years ago^3,5^. Later, the northern population diverged from the southern population about 493 generations ago (ranging from 102 to 1,490 generations in simulated datasets) (Fig. 4B). Post-hoc analyses provided goodness-of-fit for this scenario, with the original dataset well embedded in the prior PODs population and nested in the posterior PODs population (see Supporting Information Table S2 for details of this analysis).

## Discussion

Wild populations of *C. canadensis* that were sampled across its native range in the U.S. revealed high levels of genetic diversity and population differentiation, the presence of population structure, limited gene flow, and an ancient bottleneck that temporally coincides with the last glacial period in North America. We detected the presence of geographical clusters, longitudinally in the southern region (along U.S. coastal plains), and northern region. Evolutionary history analyses revealed an ancient bottleneck event occurring in the *C. canadensis* population in the south followed by divergence of the northern population from the southern population of *C. canadensis*.

When populations were compared across the ecoregion divisions from which they were collected, ecoregion designations were not associated with population structure and genetic diversity of wild populations of *C. canadensis*. Low genetic variation in *C. canadensis* across ecoregions is not surprising, given that this tree species is well-adapted to a wide range of soil types, environmental conditions and habitats, has not been constrained by any major geographical barriers, and is widespread among the eastern U.S. However, we found evidence of relatively higher genetic diversity among the northernmost collection sites (IA, IN, MI, NE, and OH, and in mid-latitude North America) that are located at the periphery of the contemporary northern range of this species. A plausible explanation for this discrepancy between northernmost samples when compared to the southern collection sites is the probability of one major refugium or admixture between small, but genetically rich, populations in refugial contact zones^58–60^. This effect is most evident in species that experience reproduction via long distance gene flow and local adaptation among sensitive individuals in the distributed population margins. However, unlike European temperate species, eastern north American species maintained high genetic diversity in northern populations^61,62^. This high level of genetic diversity in the *C. canadensis* northern population could be maintained by long-distance seed dispersal events during range expansion from post-glacial northern refugia^62,63^.

The ability of *C. canadensis* to maintain high genetic diversity can be influenced by several factors, including wide and continuous geographic distribution, an outcrossing reproductive system, and large effective population size^59,64–66^. Many other temperate tree species sustain high genetic diversity and allelic richness across a wide geographical range even in the presence of environmental stressors^11,14,58^, pressure from insect and plant pathogens^67,68^, and human disturbances^67,69,70^. A study using microsatellite loci revealed high genetic variation among five Asian *Cercis* spp., averaging 5.7 alleles per locus^71^. A recent study focused on smaller and fragmented *C. canadensis* populations also determined that trees in this species maintain high genetic diversity and allelic richness across the native range^27^, congruent with Asian *Cercis* species and several other hardwood tree species^66,72–76^.

In addition to high genetic variability, *C. canadensis* populations also display a wide range of morphological variation across diverse environmental conditions^20,26,77–79^. For example, *Cercis* leaf shape, size, surface pubescence, and other structural features were found to be strongly related to environmental factors, such as temperature and moisture content^79–81^. In *Cercis* spp., these characteristics likely originated through local adaptation to varying climatic pressure^26,82,83^. Morphological variation in *C. canadensis* has led efforts to differentiate the species into the following three varieties: *C. canadensis* var. *canadensis* L., distributed across mesophytic habitats of the eastern U.S., *C. canadensis* var. *mexicana* (Rose) M. Hopkins (Mexican redbud) and *C. canadensis* var*. texensis* (S. Watson) M. Hopkins (Texas redbud), which are both commonly found in semi-arid regions of central Mexico and southwestern Texas^26,84–86^. However, the validity of such sub-specific classification has been questioned because of the highly continuous pattern of morphological variation in *C. canadensis* populations across its range. Moreover, current phylogenetic studies were unable to provide sufficient support to validate these divisions^84,87^. Because *C. canadensis* var. *mexicana* and *C. canadensis* var. *texensis* were not represented in our study, our data will not assist in resolving this question.

Widely distributed tree species that grow in large populations usually have low genetic differentiation and have limited population structure across their geographic range^15,60,65,66,68,88^. Populations of *Viburnum rufidulum* Raf.^89^ and *Cornus florida* L.^68^ are temperate tree species that are widely distributed in the southeastern U.S. and have low genetic differentiation with weak population structures. Across populations of *V. rufidulum* and *C. florida*, high levels of gene flow via pollen and seed dispersal may have reduced genetic variability^75,89^. Contrary to these studies, high genetic differentiation observed among widely distributed populations of *C. canadensis* may be attributed to a limited gene flow, as well as the demographic history of the species.

Similar to many other self-incompatible forest tree species^20^, gene flow in *C. canadensis* is dependent upon various pollen and seed dispersal mechanisms. Flight distance of insect pollinators varies from one to several miles^90,91^, which would limit long distance gene flow by pollen dispersal among trees. Seedpods and seeds of *C. canadensis* are relatively heavy and typically fall in close proximity to the parental tree. Progeny that survive grow as non-reproductive seedlings during the next several years^57,92^ and yield half-sib “neighborhoods” within a localized spatial scale^89,93–96^. Several seed-feeding mammals, such as eastern woodrats (*Neotoma floridana* Ord)^97^, and birds including quail^98^ contribute to the dispersal of *C. canadensis* seed to some extent. Small rodents and deer may repeatedly eat from the same tree, thus carrying the closely related, half-sibling propagules (if eaten when seeds have matured) for distances restricted to the retention time of fecal scats^57,97,99–101^. To fully understand gene flow patterns and predict changes to *C. canadensis* distribution patterns, it may be helpful to unravel the seed and pollen dispersal methods and efficacy of seed transport by animals that have been associated with this tree species. However, seed transport efficacy will likely be limited to the relatively short distances traveled by these animals during foraging. Fruit consumption rate by animals is also restricted by reliance upon *C. canadensis* fruits as emergency food in late fall or winter, and this behavior would lower efficiency of functional seed dispersal^57,97,99–101^. These events likely limit the gene flow to short distances, create spatial genetic structures, and increase the likelihood of inbreeding at a local level, as revealed in fine scale level assessments of *C. canadensis*^27,69,102^. We also collected *C. canadensis* samples from New York (U.S.) that represent individuals occurring farther north than the reported geographic range of the species. These individuals also could result from open-pollinated escapes subsequent to introduction of *C. canadensis* into managed landscapes.

STRUCTURE analysis of the *C. canadensis* dataset revealed the presence of two geographically distinct clusters, designated as northern and southern clusters, that are divided longitudinally northwest by southeast along a Kentucky-Tennessee-Mississippi transition zone. From this evidence, the southern Appalachian Mountains have not posed a barrier, as populations belonging to the northern STRUCTURE clusters were found on both sides of the Appalachian Mountains. The presence of only two genetic clusters is congruent with the simple postglacial lineage theory presented for eastern North American tree species^59,103^. The most recent glacial event ended approximately 21,000 years ago. By its conclusion, boreal and temperate tree species had shifted closer to mid-latitudes within the eastern U.S., where many species survived within bottlenecked refugial populations^104^. According to our DIYABC supported scenario, a southeastern refugium was also likely to be the major postglacial refugium for *C. canadensis.* This scenario is further supported by several phylogenetic studies that have indicated that southeastern U.S. populations served as one prominent large-scale, post-glacial refugium for many temperate species^14,103,104^. Modern day temperate species including *Fagus grandifolia* Ehrh. (American beech), *Acer rubrum* L. (Red maple), and *C. florida* (Flowering dogwood), for example, likely originated from this southeastern refugium^4,14,105^. *Cercis canadensis* also shares the same geographic distribution as these temperate tree species, and modern-day wild populations of *C. canadensis* are ubiquitous throughout this region.

Our analyses also revealed support for several possible micro-refugia across the eastern U.S., evident in genetic differences among populations and presence of substructures that lack distinct centers. Several studies on different tree species indicated presence of refugia in the eastern U.S. such as southern Appalachian Mountains, southeastern coastal plains, and lower Mississippi River Valley (McLachlan et al., 2005; Potter et al. 2011). Post-glacial *C. canadensis* populations from this geographical range may have spread northward to establish the current species distribution. Post-glacial populations of other tree species from this range are adapted to semi-arid to xeric environments^9,14,103^ and present adaptive characteristics that are similarly evident in mid-western *C. canadensis* populations. Moreover, the presence of a number of refugia or fragmented refugia is also can be supported by the high genetic diversity and allelic richness of the modern-day *C. canadensis* populations^60^.

Phylogeographical studies of other tree species and animals indicate that they survived as northern cryptic micro-refugia^104,106–108^. Due to insufficient fossil data from the Late Pleistocene in the northern region, it is difficult to conclude the definite presence of northern refugia of the *C. canadensis* populations^5,6^. The best supported DIYABC evolutionary scenario suggests that at the time of the last glacial period *C. canadensis* populations persisted within a southern population group. Therefore, we also find little evidence for the possibility of a northern cryptic refugium for this species. Instead, pre-glacial *C. canadensis* was distributed in midwestern and southeastern U.S. populations, which later survived in one or more post-glacial midwestern and southeastern U.S. refugia. As a consequence of long-term population isolation within the refugial areas, a post-glacial refugial population in the midwestern U.S. may have diverged from the southeastern large refugium population, giving rise to a genetically differentiated northern spatial cluster^70,95^. Also, this post-glacial northern population may have later migrated from the midwestern U.S. to establish the current range distribution.

Ancestors of North American *Cercis* species are thought to have originated under mesic conditions and may have dispersed into North America across the North Atlantic Land Bridge^81,84,109^. According to several studies, ancestral *Cercis* population adapted to the drier environment and then spread into the Northern hemisphere during the mid-Miocene period^84,87^. It is possible, then, that this ancestral, un-sampled, mid-Miocene *Cercis* population gave rise to the southern *C. canadensis* population as suggested by DIYABC Scenario 2a.

This economically and ecologically significant deciduous shade tree species has a number of desirable morphological variations and ornamental characteristics including foliar color and texture, flower color variation, drought tolerance, pathogen resistance, as well as a wide variety of architectural forms^20,26,57^. Fruits and seeds of *C. canadensis* are consumed by several bird species and small mammals^57,97,99,101^, and many pollinators depend on this tree for an early season food source^110^. More than three dozen cultivars are available commercially and nursery stock sales of the species contribute to more than $27 M annually in the U.S.^71,111^. When paired with recent introductions of novel horticultural cultivars with highly desirable characteristics, the value of adaptive traits that are likely to exist across different geographic localities supports the importance in conserving local level diversity of *C. canadensis*. These populations are genetic reservoirs of potential variability that can provide breeding programs with the resources needed to improve selected traits (e.g., limiting seed pod productivity in landscape specimens) and provide additional opportunities for developing high-value cultivars for commercial trade. Future work should also focus on identifying important adaptive traits in wild populations that can be used to help ensure that *C. canadensis* populations will persist and will continue to adapt to a changing climate that is occurring across portions of the current species distribution.

## Data Archiving Statement

After the manuscript is accepted, data will be publicly available and deposited to Dryad Depository.

## Supplementary Information

Supplementary Information 1.

Supplementary Information 2.

Supplementary Information 3.
