# Supplementary material for: Genetic diversity in North American Cercis Canadensis reveals an ancient population bottleneck that originated after the last glacial maximum
Source: Sci Rep. 2021 Nov 8;11:21803. doi: 10.1038/s41598-021-01020-z (PMC8576035; doi:10.1038/s41598-021-01020-z)

**Supporting Information**

**Genetic diversity in North American *Cercis canadensis* reveals an ancient population bottleneck that originated after the Last Glacial Maximum**

Meher Ony, William E Klingeman, John Zobel, Robert N Trigiano, Matthew Ginzel, Marcin Nowicki, Sarah Boggess, Sydney Everhart, Denita Hadziabdic

The following Supporting Information is available for this article:

Fig. S1: For major eco-regions of *Cercis canadensis* collection zones

Fig. S2: Scatterplot of pairwise genetic vs. geographical distance of *Cercis canadensis* individuals.

Table S1: Pairwise FST matrix of Nei's genetic distance of *Cercis canadensis*.

Table S2: DIYABC scenario analyses of *Cercis canadensis* population clusters.

Fig. S1: Four major eco-regions of *Cercis canadensis* collection zones. Each color depicts a unique eco-region province. Mid and eastern United States eco-region provinces (USDA Forest Service) are divided into broad four division. The ecoregion map was generated in program R using the following packages: *ggplot2*, *dplyr*, *rgdal*, *raster*, *ggsn*, and *rworldmap*. Ecoregion provinces were visualized using the shapefiles created by USDA Forest Service and the National Atlas of the United States.


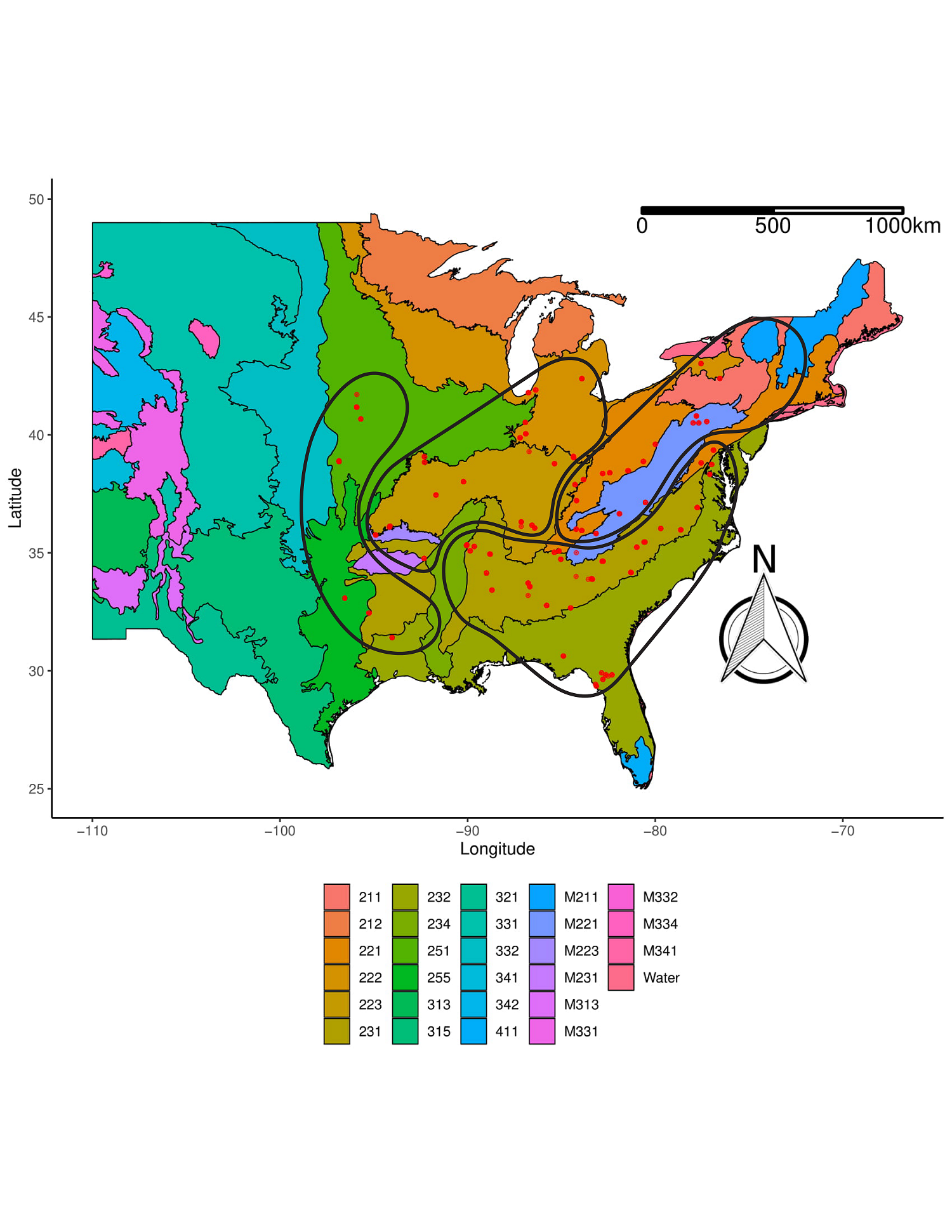


Fig. S2: Scatterplot of pairwise genetic vs. geographical distance of *Cercis canadensis* individuals. P values were calculated using Mantel tests with 10,000 randomizations. Values on the x-axis are in log scale and line indicates the best fit of least-squares regression.


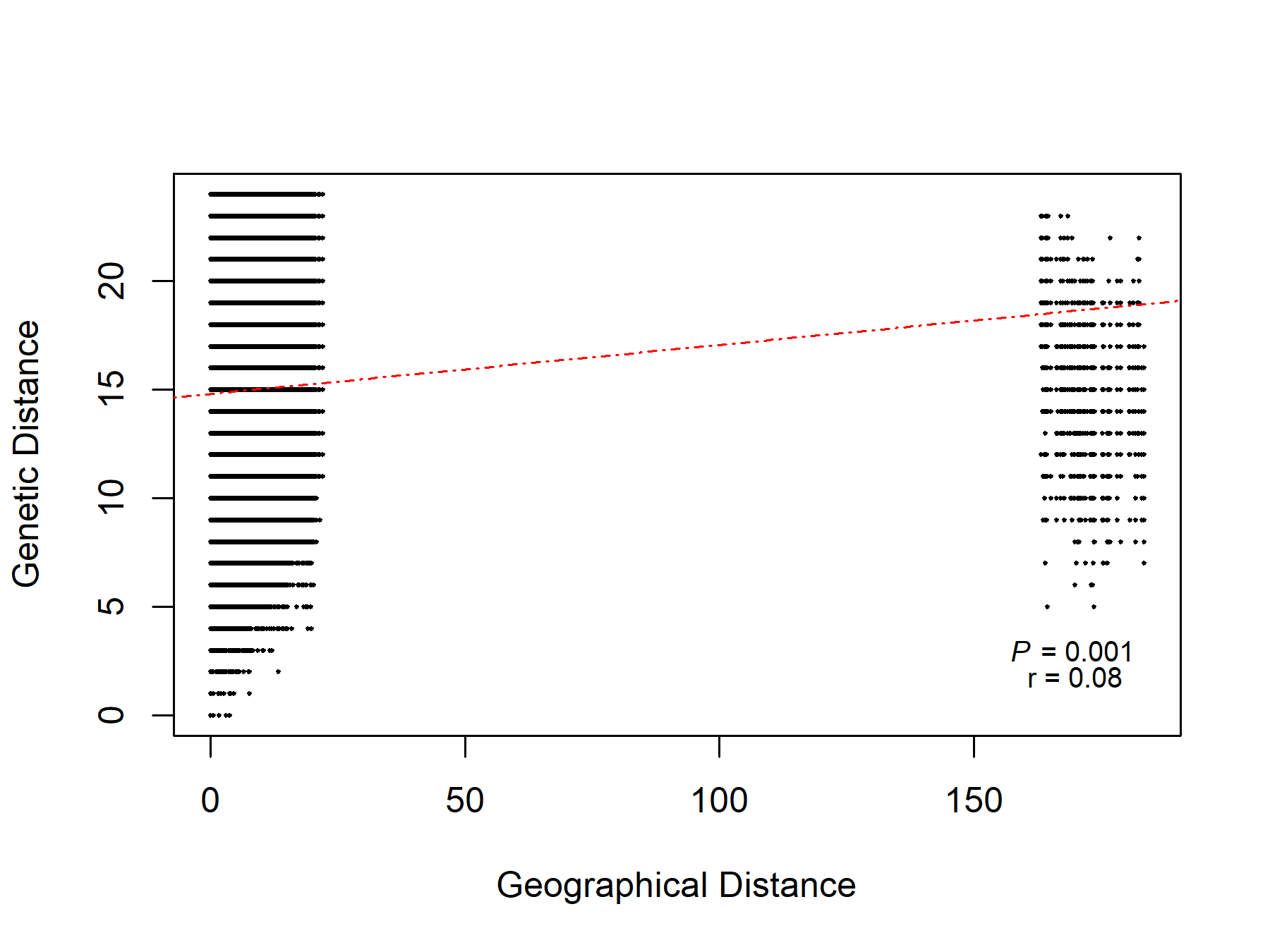

Supplement: Supplementary file 1 — Supplementary Information 1. [file 41598_2021_1020_MOESM1_ESM.docx]
